# Acute Type A Aortic Dissection With Compartment Syndrome

**DOI:** 10.1016/j.jaccas.2025.105146

**Published:** 2025-10-29

**Authors:** Juxiang Wang, Junhao Xie, Lihua Li, Hua Peng

**Affiliations:** a Department of Intensive Care Unit, Xiamen Cardiovascular Hospital, Xiamen University, Xiamen, China; b Department of Cardiac Surgery, Xiamen Cardiovascular Hospital, Xiamen University, Xiamen, China

**Keywords:** acute compartment syndrome, acute type A aortic dissection, continuous blood purification, fasciotomy, malperfusion syndrome, open repair

## Abstract

**Background:**

Malperfusion syndrome (MPS) and acute compartment syndrome (ACS) complicating acute type A aortic dissection (ATAAD) are lethal conditions. Despite the restoration of true lumen flow following successful surgical or endovascular interventions, patients may succumb to metabolic abnormalities and end-organ failure.

**Case Summary:**

This case presents a series of therapies for ATAAD accompanied by severe MPS and ACS of the lower extremity. Immediate open repair surgery was performed to prevent aortic rupture and reopen the visceral artery, and endovascular treatment ensured limb reperfusion. Fasciotomy was performed for ACS with compartment decompression, and continuous blood purification facilitated the restoration of organ function.

**Discussion:**

Proximal aortic repair is unlikely to completely restore perfusion to aortic branch vessels in cases of ischemia caused by dissection-related obstruction. A multimodal management strategy, encompassing medical therapy, urgent open repair, endovascular stenting, fasciotomy, continuous blood purification, and a multimodal team, may effectively salvage the patient.

**Take-Home Messages:**

ATAAD is an emergent and potentially fatal disease, particularly complicated by MPS. A multimodal management strategy may yield satisfactory clinical outcomes.

## Graphical Abstract

**Figure:**
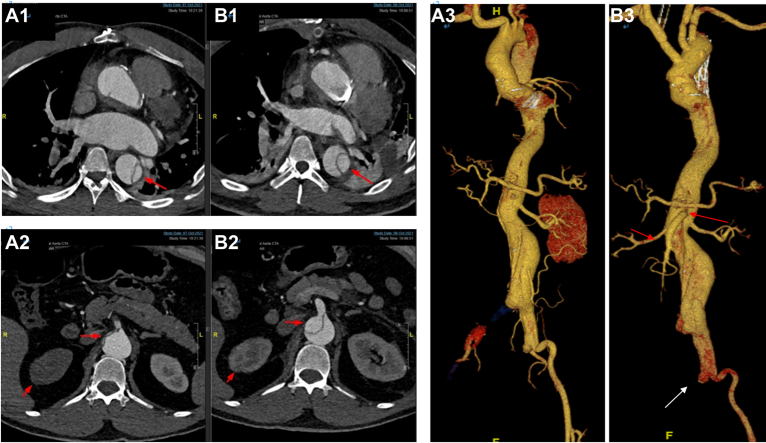

## History of Presentation

A 36-year-old man was diagnosed with Marfan syndrome and underwent a Bentall procedure along with mechanical aortic valve replacement 10 years ago. Warfarin was regularly prescribed, with a target international normalized ratio of 2.0. He had controlled hypotension and had no history of diabetes, smoking, or alcohol consumption.

**Take-Home Messages**

• Open aortic repair and endovascular reperfusion are effective therapies for patients with acute type A aortic dissection complicated by malperfusion syndrome.
• Despite the restoration of true lumen flow following open or endovascular intervention, ischemia–reperfusion injury, metabolic abnormalities, and end-organ failure following acute type A aortic dissection necessitate a multimodal management strategy and a collaborative team approach.

## Past Medical History

The patients experienced sudden chest pain and paralysis of the right lower limb while sleeping 5 hours before admission. He was conscious, with a heart rate of 92 beats per minute upon admission. The blood pressure in the affected limb was significantly lower than that in the other limbs (75/43 mm Hg vs 140-150/82-85 mm Hg). The muscle strength of the affected limb was assessed at grade 3/5, accompanied by a lower temperature. Laboratory results indicated an international normalized ratio of 1.76, creatinine level of 105 μmol/L, and creatine kinase level of 402 U/L (normal range, 40-310 U/L) upon admission. A computed tomography angiography was immediately performed, which revealed an acute type A aortic dissection (ATAAD) with different occlusions of the superior mesenteric, right renal, and right iliac arteries.

## Differential Diagnosis

The diagnoses were ATAAD, Marfan syndrome, hypertension, and history of cardiac surgery. Acute kidney injury (AKI), acute compartment syndrome (ACS), and malperfusion syndrome (MPS) require more evidence.

## Management (Medical/Interventions)

**Figure 1:**
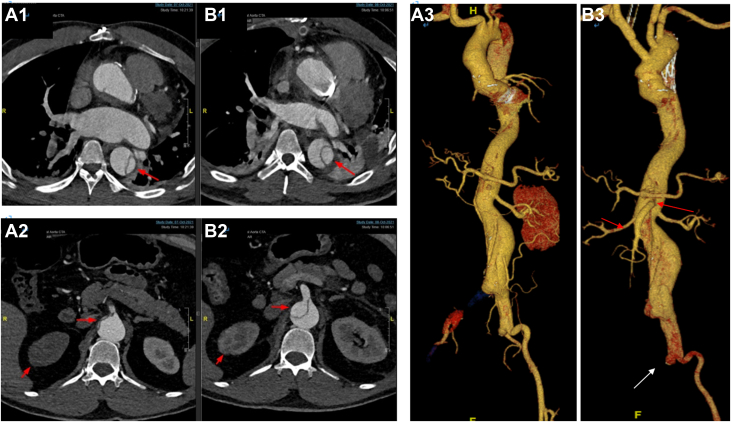
Dissection-Related Branch Vessels Before and After Open Repair (OR) Surgery (A1-A3) Computed tomography angiography results before OR. (B1-B3) Results following OR. Red arrows (A1 and B1): the true lumen is more widely opened after than before OR. Red arrows (A2 and B2): the true lumen of the superior mesenteric artery is more open after OR, with blood flow improvement, than before OR. Contrast imaging of the right kidney regained after OR. Red arrow (A3 and B3): Three-dimensional reconstruction indicate superior mesenteric and right kidney artery were visualized following OR; white arrow (B3): The right iliac artery continues to show no blood flow.

Upon diagnosis of ATAAD, treatment was immediately initiated for blood pressure, pulse rate, and pain control using β-blockers, calcium channel blockers, and analgesics. The patient underwent emergent open repair (OR) surgery for ATAAD 16 hours after symptom onset. The surgical procedure continued with ascending aortic replacement and total arch replacement using a 4-branch prosthetic graft, along with the implantation of an elephant trunk stent into the true lumen of the distal aorta lasting for 10 hours. A follow-up computed tomography angiography after OR revealed that the superior mesenteric and right renal arteries were reopened; however, no flow was observed in the right common iliac artery. The patient required mechanical support and regained consciousness 6 hours after OR, exhibiting clear consciousness without abdominal pain, abdominal distension, or bloody stool, followed by sedation and analgesia. An endovascular intervention was performed, and stents were inserted into the abdominal and right iliac arteries. The blood flow of the true lumen was restored, confirmed by intraoperative angiography 36 hours after symptom onset. Figure 1 shows the dissection-related branch vessels before and after OR.

**Figure 2:**
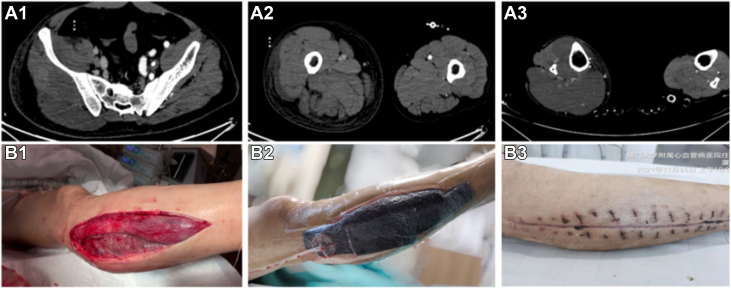
Acute Compartment Syndrome of the Right Lower Limb and Fasciotomy Decompression (A1-A3) Computed tomography showing the swelling of the right lower limb, ipsilateral thigh (A1), buttocks (A2), and waist (A3). (B1-B3) Injury and recovery of the right lower limb. Emergency fasciotomy decompression showed the anterior and lateral calf muscles displayed severely compromised blood supply (B1), vacuum-assisted wound closure (B2), and sutured wound without infection (B3).

However, the right lower limb swelling extended to the ipsilateral thigh, buttock, and waist, with creatine kinase levels reaching 59,545 U/L. The urine output decreased to 10 mL/h, whereas creatinine levels increased to 221 μmol/L. The patient exhibited marked MPS due to ATAAD, resulting in ACS of lower extremity and AKI. Prompt intervention is critical for the preservation of the limb, kidney, and life. Continuous blood-purification techniques, including continuous venovenous hemodiafiltration and hemoperfusion, were initiated 42 hours after symptom onset. The swelling of the affected limb progressed, the pulse of the dorsal artery weakened significantly, and creatine kinase levels continued to rise, reaching a peak of 68,249 U/L. As the pressure of the anterior compartment increased to 40 mm Hg, an emergency fasciotomy was performed on the lateral side of the right calf, and adequate decompression was performed in the anterior, lateral, superficial, and deep posterior compartments 60 hours after symptom onset. The calf muscles had severely compromised blood supply and severe swelling, necessitating vacuum-assisted wound closure. Figure 2 illustrates ACS and fasciotomy decompression.

## Outcome and Follow-Up

Following fasciotomy and continuous blood-purification therapy, the creatine kinase and creatine levels gradually declined. The limb wound was sutured on postoperative day 18 (Figure 2B3), showing no signs of infection. The patient underwent 8 sessions of hemoperfusion for 7 days and continuous venovenous hemodiafiltration therapy for 17 days. By day 46, serum creatinine levels decreased to 129 μmol/L, and mechanical ventilation was provided for 14 days after OR. The intensive care unit stay lasted 28 days, and the total hospital stay duration was 54 days. Muscle strength approached normal levels, and the patient could walk. However, slight abnormal sensations persisted at the time of discharge.

**Visual Summary:**
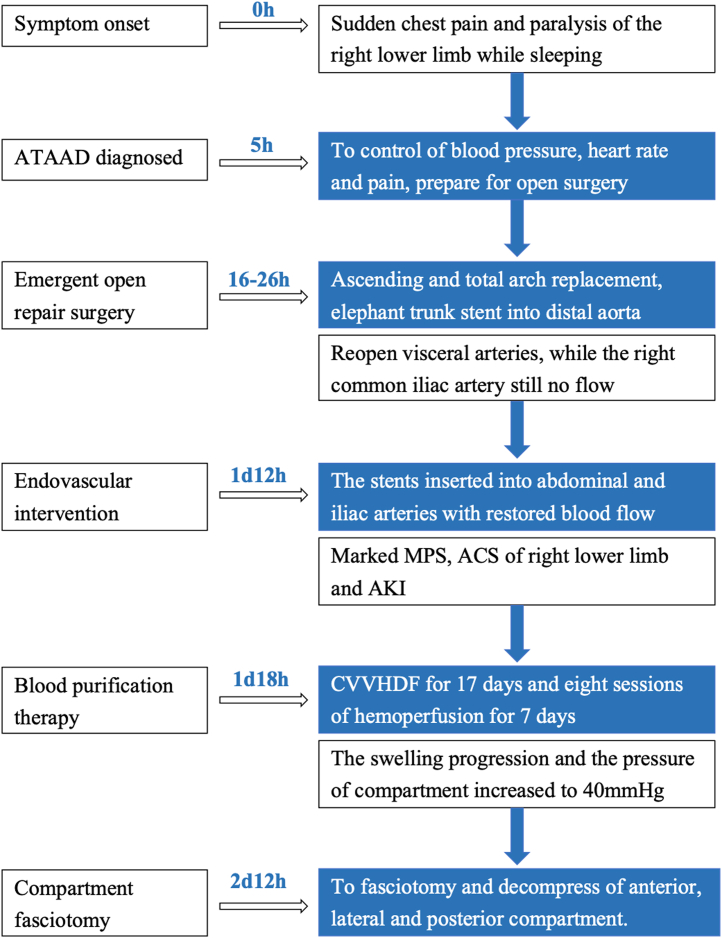
A Clinical Timeline of the Case All time points represent the time span from symptom onset. ACS = acute compartment syndrome; AKI = acute kidney injury; ATAAD = acute type A aortic dissection; CVVHDF = continuous venovenous hemodiafiltration; MPS = malperfusion syndrome.

## Discussion

Immediate OR is employed to prevent aortic rupture in patients with ATAAD^1^ and has saved numerous lives. However, MPS remains a life-threatening complication of ATAAD. Despite the rarity of peripheral malperfusion, it can be equally devastating. Given the severity and urgency of this situation, multimodal management strategies, including OR and endovascular surgery,^2^ often necessitate a multidisciplinary team approach.

Here, we report the successful management of a case of ATAAD complicated by the obstruction or occlusion of several branch vessels. The multimodal management included initial medical therapy, emergent OR and endovascular intervention, fasciotomy decompression of the compartment, blood-purification techniques, and a collaborative team effort. Initial medical therapy can optimize patient stabilization before surgical intervention. Immediate OR effectively prevents mortality associated with aortic rupture and reopens branch vessels affected by dissection. An endovascular stent was used to restore patency in the right common iliac artery, which had no blood flow after OR. Emergency fasciotomy and blood-purification techniques, including several hemoperfusion sessions,^3^, ^4^, ^5^ were employed to address ACS resulting from prolonged arterial occlusion while providing protection against AKI. These strategies aim to prevent rupture, preserve organ function, and save lives. Ultimately, the patient survived, his kidney function was restored, and the limb injury due to rhabdomyolysis was resolved, with minimal dysfunction.

## Conclusions

Patients with ATAAD complicated by MPS exhibit highest in-hospital mortality rates. However, when combined with medical therapy, urgent open cardiac and endovascular interventions, decompressive fasciotomy, and continuous blood purification, these approaches can effectively salvage the patient with ATAAD and severe ACS.

## Funding Support and Author Disclosures

The authors have reported that they have no relationships relevant to the contents of this paper to disclose.
